# Hydro-chemical based assessment of groundwater vulnerability in the Holocene multi-aquifers of Ganges delta

**DOI:** 10.1038/s41598-024-51917-8

**Published:** 2024-01-13

**Authors:** Asish Saha, Subodh Chandra Pal, Abu Reza Md. Towfiqul Islam, Aznarul Islam, Edris Alam, Md. Kamrul Islam

**Affiliations:** 1 Department of Geography, The University of Burdwan, Purba Bardhaman, West Bengal 713104 India; 2 Department of Disaster Management, Begum Rokeya University, Rangpur, 5400 Bangladesh; 3 Department of Development Studies, Daffodil International University, Dhaka, 1216 Bangladesh; 4 Department of Geography, Aliah University, 17 Gorachand Road, Kolkata, 700014 India; 5 Faculty of Resilience, Rabdan Academy, 22401 Abu Dhabi, United Arab Emirates; 6 Department of Geography and Environmental Studies, University of Chittagong, Chittagong, 4331 Bangladesh; 7 Department of Civil and Environmental Engineering College of Engineering, King Faisal University, 31982 AlAhsa, Saudi Arabia

**Keywords:** Environmental impact, Environmental impact, Sustainability

## Abstract

Determining the degree of high groundwater arsenic (As) and fluoride (F^−^) risk is crucial for successful groundwater management and protection of public health, as elevated contamination in groundwater poses a risk to the environment and human health. It is a fact that several non-point sources of pollutants contaminate the groundwater of the multi-aquifers of the Ganges delta. This study used logistic regression (LR), random forest (RF) and artificial neural network (ANN) machine learning algorithm to evaluate groundwater vulnerability in the Holocene multi-layered aquifers of Ganges delta, which is part of the Indo-Bangladesh region. Fifteen hydro-chemical data were used for modelling purposes and sophisticated statistical tests were carried out to check the dataset regarding their dependent relationships. ANN performed best with an AUC of 0.902 in the validation dataset and prepared a groundwater vulnerability map accordingly. The spatial distribution of the vulnerability map indicates that eastern and some isolated south-eastern and central middle portions are very vulnerable in terms of As and F^−^ concentration. The overall prediction demonstrates that 29% of the areal coverage of the Ganges delta is very vulnerable to As and F^−^ contents. Finally, this study discusses major contamination categories, rising security issues, and problems related to groundwater quality globally. Henceforth, groundwater quality monitoring must be significantly improved to successfully detect and reduce hazards to groundwater from past, present, and future contamination.

## Introduction

Groundwater is a crucial source of drinking water, and its availability is essential for economic growth in urban and rural areas worldwide^1,2^. Groundwater is less vulnerable to contamination and pollution than surface water and is widely used for domestic purposes^3,4^. Due to its high percentage, reduced sensitivity to pollution, and large storage capacity, groundwater is more important than surface water at a socioeconomic level worldwide. Groundwater undergoes a natural filtration process that removes bacteria and odors, making it suitable for drinking^5^. Groundwater has many advantages, including meeting water supply needs for industrial, agricultural, and other sectors. In many parts of the world, groundwater is the primary source of fresh water with 50% of portable water demands being met by groundwater, 40% of which is used for industry, and the remaining portion used for irrigation^6^. As the world's population grows, its dependence on groundwater also increases, with 33% of people depending on it to meet their daily needs^7,8^. Unfortunately, more groundwater is being consumed than replenished or recharged, which stresses on the availability of this precious natural resource. As a result, groundwater overuse has led to declining water tables, declining water quality, and ongoing frequent land subsidence activities^9^.

Despite all the advantages of groundwater, many nations, especially developing countries like India and Bangladesh, are quickly experiencing a crisis of diminishing groundwater quality due to misuse and contamination^10^. Groundwater contamination can result from both natural and human causes. However, the quantity and groundwater quality are highly vulnerable today. Anthropogenic activities have accelerated the rate at which the quality of groundwater is declining. Unplanned land-use activities owing to industrialization and subsequent urbanization have led to rising groundwater contamination in recent decades^11^. Urbanization increases impervious surfaces, worsens ephemeral runoff, increases flood risk, and reduces subsequent groundwater recharge. In addition, saltwater intrusion exacerbates the situation in coastal areas, severely threatening the city's water supply and lowering the living standards of residential homes^12^. Human activities such as irrigation and climate change are currently impacting groundwater quality and increasing its sensitivity to contamination on a broader scale^13^. Chemical fertilizers are exacerbating the critical issue of nitrate poisoning of aquifers^14^.

One of the largest natural groundwater catastrophes for humanity has been reported to be arsenic (As) pollution in the groundwater. A study revealed that only five Asian nations, namely “Taiwan, China, India, Bangladesh, and Thailand”, were acknowledged as having groundwater contamination due to As in the late twentieth century^15^. At least 100,000 individuals in these impacted nations are exposed to As poisoning through their drinking water. In India, several places in the Brahmaputra and Ganges River’s floodplain have been affected by groundwater contamination with arsenic at levels higher than the permitted limit of 10 µg/L^16^. Currently, Bangladesh and India have the highest number of As contaminated areas and associated health issues^17^. Specifically, the Ganges delta of Indo-Bangladesh region is highly affected by groundwater contamination due to As^14,18,19^. These floodplains are made of recent alluvial aquifers from the Holocene period that originated in the Himalayan region^20^. Consequently, people in these afflicted areas have been regularly exposed to drinking water from hand tube wells contaminated with arsenic. In Bangladesh, using contaminated surface water resources, such as, ponds, rivers, and shallow dug-wells has led to water-borne illnesses like cholera, diarrhea, and dysentery^21^. Recent estimates suggest that 5 million people in West Bengal's North 24 Paraganas district consume water with an arsenic concentration of more than 50 µg/l and As rich causing approximately 50,000 people to develop skin sores in West Bengal. The population impacted in the geographic area of the issue are alarmingly growing each year^22^. Moreover, As has also entered the food chain through rice (paddy) production in the Indo-Gangetic plains via irrigation water carrying As^23^.

From a methodological viewpoint, statistical, machine learning (ML), and artificial intelligence have been utilized to evaluate groundwater vulnerability globally. ML algorithms offer several advantages over statistical methods, as they can efficiently analyze large datasets^24,25^. Additionally, geospatial approaches provide quick, efficient spatial, temporal, and spectral analysis of data over a wide area^26^. As a result, numerous researchers have combined geospatial technology with ML algorithms, such as the deep learning network used by Elzain et al.^27^ in south Korea, the BRT model used by^28^ in Iran, RF used by Pal et al.^14^ in coastal areas of West Bengal, and the Bayesian model averaging (BMA) used by Gharekhani et al.^29^ in West Azerbaijan, Iran, to assess groundwater vulnerability.

Considering the ongoing phenomena related to groundwater resources worldwide, particularly in the Ganges delta, very few studies have been undertaken that couple hydrochemical factors with ML algorithms^30–32^. Literature review on groundwater vulnerability highlights that the Ganga–Brahmaputra delta in the Indo-Bangladesh region stands out as a significant area globally affected by arsenic contamination^17,33^. The widespread use of tube wells for water supply in the Ganges Delta is a critical concern, leading to severe arsenic poisoning. In addition to arsenic, some regions in India also face challenges with elevated fluoride levels in groundwater. The Ganges Delta, marked by high population density and robust agricultural activity, necessitates sustainable water resource management to ensure optimal utilization. Hence, our study focuses on assessing groundwater vulnerability in the Ganges delta, emphasizing the urgency of effective water resource management in this crucial region. Therefore, researching groundwater vulnerability in this area is crucial for managing groundwater effectively and making it safe for consumption. In this regard, the presents study uses geospatial techniques and ML algorithms, including the LR, RF, and ANN models, to evaluate groundwater vulnerability in the study region. The distinctive aspect of this research resides in integrating statistical, ML, and neural network algorithms with hydrochemical factors. This fusion aims to comprehend the fluctuations in modeling outcomes and their corresponding spatial distribution in such a vast region. Furthermore, quality assessment for irrigation water in this study region has been assessed using USSL and Wilcox’s diagram. This study has distinctive contribution in its novel outcomes and insights, which contribute optimal perspectives to the current body of existing literatures. Furthermore, the research insight into regional disparities, illuminating differences or distinctions within a specific geographical area. The outcomes of this study will be helpful to environmentalists and policy-makers in planning for the local people regarding the safe consumption of water resources.

## Study area

**Figure 1:**
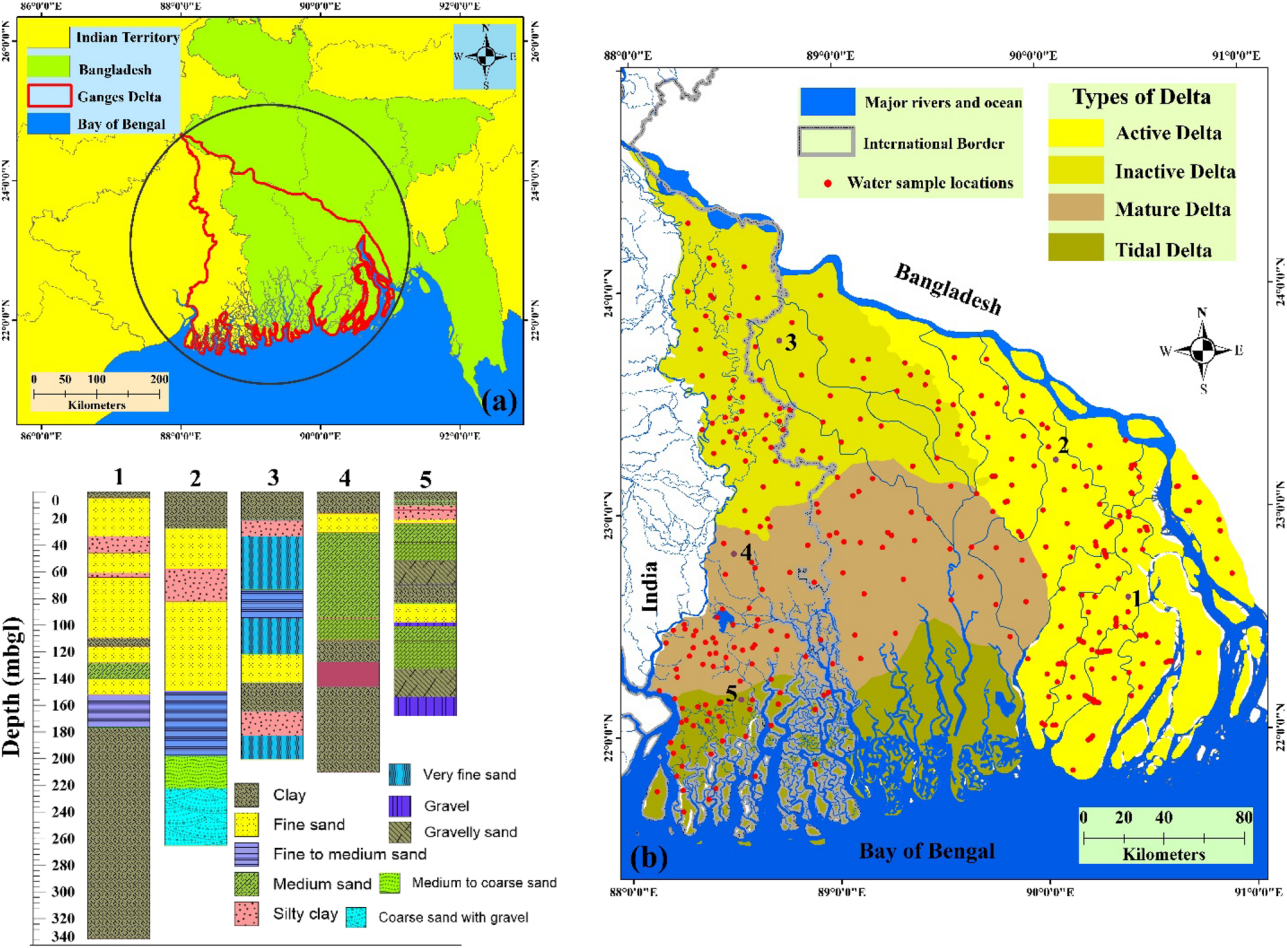
Details about the study area: (**a**) Ganges delta in a transnational boundary of Indo-Bangladesh region, (**b**) Ganges delta and its morphological types and (**c**) litholog profile of some selected points (this map was generated using ArcGIS, version: 10.3.1, www.esri.com/arcgis).

The Ganges and Brahmaputra delta, known as the Ganges delta, is one of the mega-deltas in the world, covering an area of approximately 105,000 km^2^. It consists of Bangladesh and parts of India’s state of West Bengal, formed by sedimentation of the Ganga, Meghna and Brahmaputra rivers at the Bay of Bengal during the late Holocene to recent times^20^. The delta stretches from 21° 10′ 42″ to 24° 50′ 39″ N latitude and 87° 30′ 21″ to 91° 26′ 46″ E longitude (Fig. 1) and has a shoreline of nearly 350 km along the Bay of Bengal. The Ganges delta has been divided into three parts from a geological perspective i.e., “Moribund delta, Active delta and Mature delta”^34^. The delta's stratigraphic section shows alternating sand-dominated and fine-grained phases with intricate interfingerings between them^33^. This delta enclosed by “Precambrian crystalline rocks” to the north and west and the “Assam-Arakan Neogene fold belt” to the east, signifies a comprehensive sedimentation history during the late Quaternary period^35^. Literatures indicates that numerous elevated terraces from the Pleistocene era are present both within and along the periphery of its alluvial plain^36^. Present evidence considering remote sensing supporting neotectonics activities in the Gangetic plain^37^. Salinity has impacted aquifers in the coastal regions of Bangladesh, reaching depths of up to 350 m, furthermore the salinity levels in the upper aquifers of the coastal region, reaching depths of 200–250 m, demonstrate notable fluctuations and experience abrupt changes over short distances^38^. The monsoon season (June–October) accounts for more than 80% of the annual rainfall, which ranges from 1500 to 2000 mm^39^. During the monsoon months, high rainfall and frequent tropical cyclones cause catastrophic flooding and saltwater intrusion in the land areas. The minimum seasonal temperature of the region varies from 12 to 24 °C, and the maximum ranges from 25 to 35 °C. The area has the largest population density compared to other deltaic regions due to the high soil fertility^30^. The Sundarbans, the world's largest mangrove forest, covers the southernmost part of this deltaic region also known as the Sunderban delta. Borehole data indicates that sediment primarily consists of sand and clay types.

## Materials and methods

### Methodology

In this study, the following methodological steps have been followed to fulfill the current research objectives:

- In the initial stage, 352 water samples were collected from the existing tube-wells in the field to assess different hydro-geochemical properties. Additionally, 352 non-sample points were created for modelling purposes. Furthermore, it is necessary to divide the dataset into train and test to assess how well machine learning models are performed. Where, train dataset is used to fit the model and the test dataset is used for validation of the respective model. The entire dataset was split into two categories in a “70:30 ratio for training and validation” of the respective models.
- A total of fifteen hydro-geochemical parameters were identified for modelling groundwater vulnerability. These parameters are “Depth (m), pH, EC (μS/cm), Salinity (ppt), Ca^2+^ (mg/l), Mg^2+^ (mg/l), Na^+^ (mg/l), K^+^ (mg/l), Cl^−^ (mg/l), HCO_3_^−^ (mg/l), NO_3_^−^ (mg/l), SO_4_^2−^ (mg/l), PO_4_^2−^ (mg/l), F^−^ (mg/l), As (μg/l)”.
- Statistical analysis, including “Pearson’s correlation coefficient, principal component analysis (PCA) and multicollinearity (MC)” test, was conducted to understand the nature of data.
- Statistical, ML and neural network algorithms i.e., “logistic regression (LR), random forest (RF) and artificial neural network (ANN)” were used for groundwater vulnerability assessment.
- Statistical evaluation metrices, such as “sensitivity, specificity, AUC-ROC, F score, Kappa coefficient, and graphical measures such as the Taylor diagram” were used to optimize the assessment of modelling output.
- “USSL and Wilcox’s diagram” used to assess groundwater quality.

The following sub-section described in details regarding the methods used in this study.

### Sampling and inventory dataset

Field-based water sample collection was the primary task to prepare several hydro-chemical parameters for assessing groundwater vulnerability. In this regard, a “random stratified” sampling method was used to collect water sample across the study region. A total of 352 water samples were collected to prepare the inventory dataset (Fig. 1). Standard procedures were followed during the collection of water samples. Sampling was done by running wells for 5 min as it removes the stagnant water from bore wells as well as hand pumps. The sample tube well was kept pumping until the pH and EC achieved stable conditions. Two independent (dry and clean) sample kits were used each with its own collection methods and safety measures, to keep the water samples that were taken. In order to transport each water sample from the field to the lab and keep it at 4 °C, we stored it in a water sample kit during sample collection. Measurements were made to analyze the groundwater samples obtained both on-site and off-site. The analyzed samples were split into two categories on the ArcGIS 10.4.1 platform based on a ratio of 70:30. The sample was used for training (70%) while the other was used for validation (30%).

In our current research, we opted for dry season (March–early June) data to model and map groundwater vulnerability in this susceptible region, excluding wet season data. Existing literature indicates a prevalent use of dry season data in studies related to arsenic-induced vulnerability studies^40^, as it is deemed more suitable for assessing vulnerability to arsenic-related risks. In the wet season, groundwater contamination occurs through the percolation and infiltration of surface water, facilitated by ample rainfall. This leads to the transfer of various particles, metals, and ions from surface water bodies to groundwater, resulting in temporary water contamination, which is not ideal for assessing water-related health hazards. In contrast, during the dry season, water levels remain normal, and there is no risk of water contamination through surface metals or other substances. Therefore, based on these considerations, we have exclusively utilized dry season data in our study.

### MC test

To ensure the accuracy of the model’s output, it is crucial to select appropriate parameters for any vulnerability assessment. To achieve this, MC analysis is one of the most important techniques. Correlation analysis has shown that a link between two or more input variables can create deviations. “Tolerance (TOL) and Variance Inflation Factor (VIF)” are two statistical measures often used to test multi-collinearity among distinct components. The predictor variables have a high degree of multicollinearity when the “TOL value is < 0.10 and the VIF value is > 5”. If the MC result exceeds this limit, the highly correlated factors are not suitable for modelling purposes and should be removed from the dataset; otherwise, the output result will not be optimal. The equations for TOL and VIF are presented below:

1 $${\text{TOL}}=1-{{\text{R}}}_{{\text{j}}}^{2}$$

2 $${\text{VIF}}=\frac{1}{{\text{TOL}}}$$

where ${{\text{R}}}_{{\text{j}}}^{2}$ is the R-squared value of regression using the j on all other variables regression model.

### Adopted methods for groundwater vulnerability modelling

#### LR

One can create a multivariate regression relationship between a dependent variable and several independent factors using LR. LR is a multivariate analysis model that can be used to forecast the existence or absence of a characteristic or result based on the values of many response variables. Many studies used LR as a standard or conventional way to verify the effectiveness of a new algorithm in vulnerability studies. The benefit of LR is that, unlike traditional linear regression, where the variables must all have normal distributions, it can use any combination of continuous and discrete variables as well as appropriate link functions^41^. The challenge in conducting vulnerability analysis using a LR model is choosing the appropriate sample size for the dependent and independent variables^42^. The components in multi-regression analysis must be numerical, and the variables in discriminant analysis, a related statistical model, should have a normal distribution. After converting the dependent variable into a logit variable, the LR procedure uses maximum likelihood estimation^43^. This is how LR calculates the likelihood of a specific event occurring^44^. The fundamental idea behind LR is investigating a problem in which a result assessed using dichotomous variables i.e., true or false (0 and 1) is determined based on a single or a series of independent factors^45^. The LR can expressed by the following equation:

3 $$f(z)=\frac{1}{1+{e}^{-z}}$$

where $z$ indicates a linear combination of a constant and the independent variables’ product, and their corresponding coefficients. The value of z varies from − ∞ to ∞, subsequently f(z) ranges from 0 to 1”:

4 $${\text{z}}=\mathrm{\alpha }+{\upbeta }_{1}{{\text{X}}}_{1}+{\upbeta }_{2}{{\text{X}}}_{2}+\dots \mathrm{\beta nXn}$$

where $\mathrm{\alpha }$ indicates constant; ${\upbeta }_{1}, {\upbeta }_{2},\dots \mathrm{ \beta n}$ represent the coefficients and ${{\text{X}}}_{1}, {{\text{X}}}_{2}, \dots {\text{Xn}}$ are the independent variables”.

#### RF

The RF model is a reliable AI method for classifying various natural hazards, including groundwater vulnerability. Breiman^46^ proposed a potent ensemble-learning method called random forest, which is one of the most widely used classifier ensemble techniques for feature selection, regression, and classification applications. RF is a tree-based ensemble learning technique that builds several decision trees while constructing models. Each tree structure in the ensemble model uses the original input data to train a bootstrapped sample^47^. Decision trees use a collection of binary rules to select a target variable. The data used to train the model comprises the target variable being predicted and a set of predictor variables. Using the predictor variables, the decision tree divides the data into homogenous datasets based on the target variable. The programme then assesses each predictor variable's ability to categorize the predicted value into the two groups. The splitting process continues until there are no more splits to be made^48^. RF prediction is viewed as the unweighted majority of class votes when solving classification issues. The bagging approach is used to select random samples of variables as part of the training dataset for model calibration^49^. The algorithm for RF is expressed as follows:

5 $$h\left(x,{i}_{k}\right),k=\mathrm{1,2},\dots n$$

where ${i}_{k}$ represents flood occurrence conditioning factors; 1, 2,…n are input vector *x*.

In a RF the general errors can be defined as follows:

6 $$GE={P}_{x,y}\left(mg\left(x,y\right)<0\right)$$

where *x* and *y* indicate the different flood occurrence conditioning factors, and *mg* represents the margin function. Again, margin function” can be described as follows

7 $$mg\left(x,y\right)={av}_{k}I\left({h}_{k}\left(x\right)=y\right)-{max}_{j\ne i}{av}_{k}I\left({h}_{k}\left(x\right)=j\right)$$

#### ANN

The ANN is a computational method that can obtain, display, and compute mapping from one multivariate data space to another. The objective of the ANN model is to provide a technique for forecasting results from inputs that have not been used in the modelling process^50^. An artificial neural network is trained using a series of examples of related input and output values. The goal of an artificial neural network is to create a model of the data-generation process in order to generalize and predict outcomes from inputs that it has never seen before. Back-propagation learning is the neural network approach that is most often utilized in the ANN model^51^. This neural network learning technique has three levels: an input layer, hidden layers, and an output layer. The network is trained using the back-propagation technique until a predetermined minimal error between the network's desired and actual output values is reached. When training is complete, the network is utilized as a feed-forward structure to provide a classification for the entire database (Paola and Schowengerdt^52^). The ANN assigns each input element a specific weight, multiplies the results, adds them up, and then uses a nonlinear transfer function to construct the outcomes. The back propagation of the ANN model is expressed by the following equations:

8 $${net}_{j}^{l}\left(t\right)=\sum_{i=o}^{p}({y}_{i}^{i-1}\left(t\right){w}_{ji}^{l}(t))$$

The net input of jth neuron of layer l and I iteration

9 $${y}_{j}^{l}\left(t\right)=f({net}_{j}^{\left(l\right)}\left(t\right)$$

10 $$f\left(net\right)=\frac{1}{1+{e}^{(-net)}}$$

11 $${e}_{j}\left(t\right)={c}_{j}\left(t\right)-{a}_{j}\left(t\right)$$

12 $${\delta }_{j}^{l}\left(t\right)={e}_{j}^{l}\left(t\right){a}_{j}\left(t\right)\left[1-{a}_{j}x\left(t\right)\right]$$

$\delta$ Factor for neuron jth in the output layer ith

13 $${\delta }_{j}^{l}\left(t\right)={y}_{j}^{l}\left(t\right)\left[1-{y}_{j}\left(t\right)\right]\sum {\delta }_{j}^{l}\left(t\right){w}_{kj}^{\left(l+1\right)}\left(t\right)$$

$\delta$ factor for neuron jth in the hidden layer ith

14 $${w}_{ji}^{l}\left(t+1\right)={w}_{ji}^{l}\left(t\right)+\alpha \left[{w}_{ji}^{l}\left(t\right)-{w}_{ji}^{l}\left(t-1\right)\right]+n{\delta }_{j}^{\left(l\right)}\left(t\right){y}_{j}^{\left(l-1\right)}\left(t\right)$$

where $\alpha$ is the momentum rate and $n$ is the learning rate within this model.

### Selected evaluation measures

Evaluating a model's performance, which establishes whether it is relevant or not, is one of the key goals of model comparison. In the geoscientific discipline, assessment metrics for applied models are crucial to estimating their best-case performance in making predictions, especially for modelling approaches based on machine learning. Henceforth, several evaluation measures have been used by many researchers in different fields of study to optimally assess the modelling output^14,24,53,54^. After a rigorous literature survey, five prevalent evaluations metrics i.e., “sensitivity, specificity, PPV, NPV, ROC-AUC, Kappa-coefficient and F-score”, were selected for this study. Alongside, the Taylor diagram is also applied in this study, which is a graphical representation of evaluation measures expressing the relationship. A useful tool for displaying and assessing classifiers is the “receiver operating characteristics (ROC) curve” the common name for a performance indicator for classification problems at different threshold levels is the AUC-ROC curve. The ROC curve, which is a graph based on the true positive rate (sensitivity) and the false positive rate (1-specificity), may be thought of as a statistic that measures how well the model performed overall^55^. The AUC-ROC value ranged from 0 to 1 and indicates a poor and good performance accordingly^56^. The following formulas were used to create the performance evaluation criteria for this study:

15 $$Sensitivity= {\text{TP}}/({\text{TP}}+{\text{FN}})$$

16 $$Specificity= {\text{TN}}/({\text{FP}}+{\text{TN}})$$

17 $$PPV=\frac{TP}{FP+TP}$$

18 $$NPV=\frac{TN}{TN+FN}$$

19 $$AUC=\frac{(\sum TP+\sum TN)}{(P+N)}$$

20 $$Precession=\frac{{\text{TP}}}{{\text{TP}}+{\text{FP}}}$$

21 $$Recall=\frac{{\text{TP}}}{{\text{TP}}+{\text{FN}}}$$

22 $$F score=2* \frac{Precession*Recall}{Precession+Recall}$$

23 $$k= \frac{{P}_{o}-{P}_{e}}{1-{P}_{e}}$$

Here, “TP is true positive, TN is true negative, FN is false negative, FP is false positive, and kappa coefficient is represented by k, observed samples by ${P}_{o}$ and predicted result by ${P}_{e}$”.

## Result

### Statistical measures of selected hydrochemical parameters

**Figure 2:**
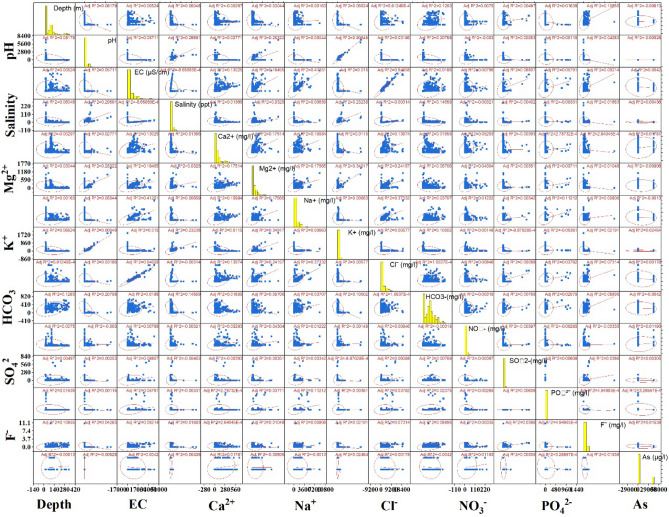
Pearson’s correlationship among the factors.

**Figure 3:**
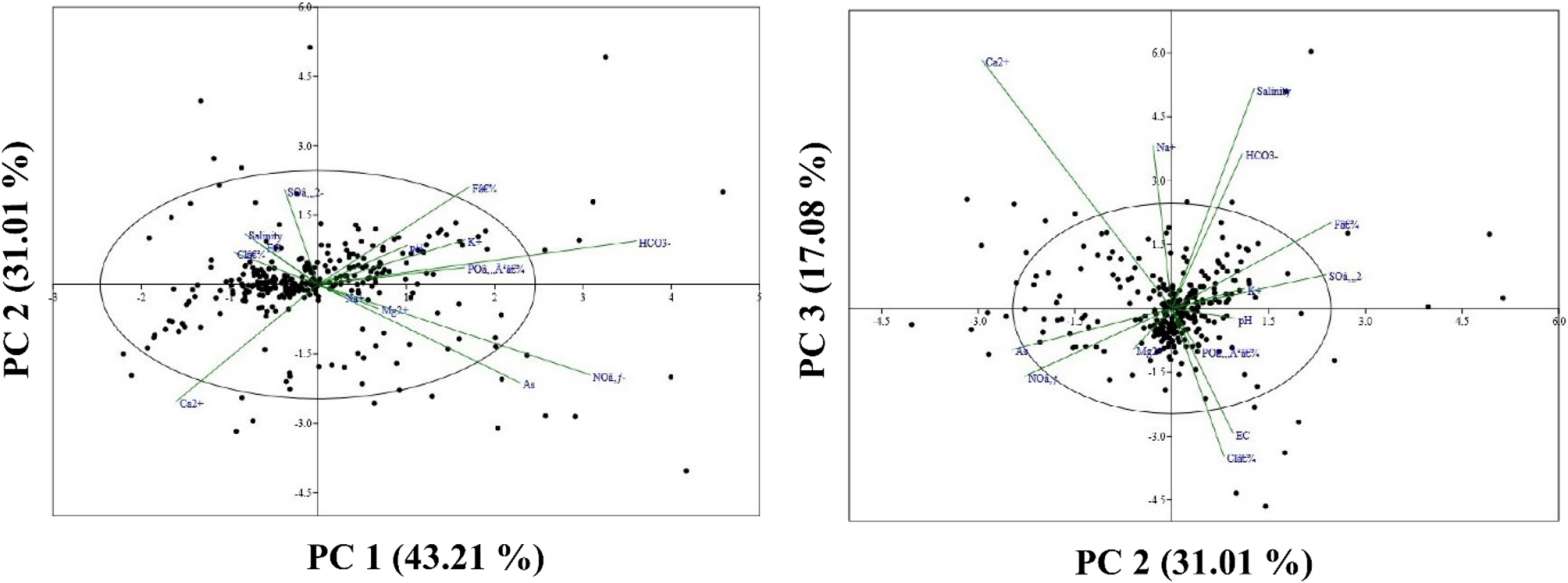
Bi-plot among the applied hydro-chemical factors.

In this study, three statistical tests were conducted on the selected hydrochemical dataset: MC, correlation coefficient, and PCA. The MC test (Table 1) showed that all factors were within the threshold value of MC, and therefore suitable for modelling purposes., The depth factor had the highest TOL and lowest VIF (0.66 and 1.515 respectively), while the Ca^2+^ factor had the lowest TOL and highest VIF (0.38 and 0.632 respectively). Pearson’s correlation coefficient was used to understand the nature of the substantial association between physical and chemical properties. The correlation coefficient (r) ranges from − 1 to + 1, with values of 0.5, 0.5–0.8 and 0.8 indicating weak, moderate, and strongly correlation, respectively. The highest correlation values were found between pH and K^+^ (0.952) and EC and CI (0.973), while moderate relationships were found between pH and salinity (0.546), pH and Mg^2+^ (0.506), EC and Na^+^ (0.644), Mg^2+^ and K^+^ (0.593), Na^+^ and CI (0.613), and the lowest values were found between Ca^2+^ and Mg^2+^ (0.422), EC and Ca^2+^ (0.365), depth and HCO_3_^−^ (0.359), etc. Details about the correlation coefficient map and table are presented in Fig. 2 and Table 2. PCA analysis showed that PC 1 consisted of 43.21% eigenvalue, followed by PC 2 and PC 3, which had 31.02% and 17.08% eigenvalue, respectively. In PC 1, the dominant factors were EC (0.933), salinity (0.927), Mg^2+^ (0.874) and CI (0.924), while important factors in PC 2 important factors were F^−^ (0.765), As (0.599) and HCO_3-_ (0.582) and in PC 3 dominant factors were PO_4_^2−^ (0.620), NO_3_ (0.582) and K^+^ (0.339). The biplot map of PC 1, PC 2 and PC 3 is presented in Fig. 3.

**Table 1:** Multi-collinearity analysis of selected factors.

Factors	VIF	TOL
Depth (m)	1.515	0.66
pH	2.564	0.39
EC (μS/cm)	2.041	0.49
Salinity (ppt)	1.852	0.54
Ca^2+^ (mg/l)	2.632	0.38
Mg^2+^ (mg/l)	1.818	0.55
Na^+^ (mg/l)	2.128	0.47
K^+^ (mg/l)	2.564	0.39
Cl^−^ (mg/l)	1.852	0.54
HCO_3_^−^ (mg/l)	2.381	0.42
NO_3_^−^ (mg/l)	2.778	0.36
SO_4_^2−^ (mg/l)	1.852	0.54
PO_4_^2−^ (mg/l)	2.083	0.48
F^−^ (mg/l)	1.724	0.58
As (μg/l)	2.326	0.43

**Table 2:** Correlationship test among the selected factors.

Correlations																
Factors	Depth (m)	Temperature (°C)	pH	EC (μS/cm)	Salinity (ppt)	Ca^2+^ (mg/l)	Mg^2+^ (mg/l)	Na^+^ (mg/l)	K^+^ (mg/l)	Cl^−^ (mg/l)	HCO_3_^−^ (mg/l)	NO_3_^−^ (mg/l)	SO_4_^2−^ (mg/l)	PO_4_^2−^ (mg/l)	F^−^ (mg/l)	As (μg/l)
Depth	1	0.192**	− 0.048	0.044	− 0.014	− 0.009	− 0.011	0.040	0.025	0.044	0.033	− 0.063	− 0.009	0.015	0.414**	0.027
pH	− 0.048	− 0.102*	1	− 0.264**	− 0.299**	− 0.213**	− 0.271**	− 0.329**	− 0.108*	− 0.300**	0.172**	− 0.068	− 0.054	− 0.012	0.046	− 0.164**
EC	0.044	− 0.157**	− 0.264**	1	0.835**	0.251**	0.551**	0.541**	0.384**	0.842**	0.016	0.250**	0.143**	0.087*	0.239**	0.176**
Salinity	− 0.014	0.079	− 0.299**	0.835**	1	0.303**	0.584**	0.615**	0.237**	0.861**	− 0.014	0.294**	0.101*	0.092*	0.187**	0.188**
Ca^2+^	− 0.009	0.071	− 0.213**	0.251**	0.303**	1	0.366**	0.302**	0.142**	0.313**	− 0.273**	0.055	0.169**	− 0.088*	− 0.040	0.075
Mg^2+^	− 0.011	0.164**	− 0.271**	0.551**	0.584**	0.366**	1	0.587**	0.264**	0.675**	− 0.106*	0.112**	0.146**	0.095*	− 0.061	0.213**
Na^+^	0.040	0.129**	− 0.329**	0.541**	0.615**	0.302**	0.587**	1	0.267**	0.633**	0.011	0.239**	0.127**	0.130**	− 0.001	0.289**
K^+^	0.025	0.132**	− 0.108*	0.384**	0.237**	0.142**	0.264**	0.267**	1	0.469**	− 0.125**	0.180**	− 0.040	− 0.041	0.059	0.027
Cl^−^	0.044	0.168**	− 0.300**	0.842**	0.861**	0.313**	0.675**	0.633**	0.469**	1	− 0.088*	0.280**	0.178**	0.083	0.192**	0.204**
HCO_3_^−^	0.033	− 0.022	0.172**	0.016	− 0.014	− 0.273**	− 0.106*	0.011	− 0.125**	− 0.088*	1	0.001	0.014	0.179**	0.197**	− 0.061
NO_3_^−^	− 0.063	− 0.029	− 0.068	0.250**	0.294**	0.055	0.112**	0.239**	0.180**	0.280**	0.001	1	− 0.029	0.049	− 0.035	0.010
SO_4_^2−^	− 0.009	0.010	− 0.054	0.143**	0.101*	0.169**	0.146**	0.127**	− 0.040	0.178**	0.014	− 0.029	1	− 0.031	0.130**	− 0.006
PO_4_^2−^	0.015	− 0.005	− 0.012	0.087*	0.092*	− 0.088*	0.095*	0.130**	− 0.041	0.083	0.179**	0.049	− 0.031	1	0.005	0.192**
F^−^	0.414**	0.125**	0.046	0.239**	0.187**	− 0.040	− 0.061	− 0.001	0.059	0.192**	0.197**	− 0.035	0.130**	0.005	1	− 0.104*
As	0.027	0.019	− 0.164**	0.176**	0.188**	0.075	0.213**	0.289**	0.027	0.204**	− 0.061	0.010	− 0.006	0.192**	− 0.104*	1

**Correlation is significant at the 0.01 level (2-tailed).

*Correlation is significant at the 0.05 level (2-tailed).

### Assessment of groundwater vulnerability

**Figure 4:**
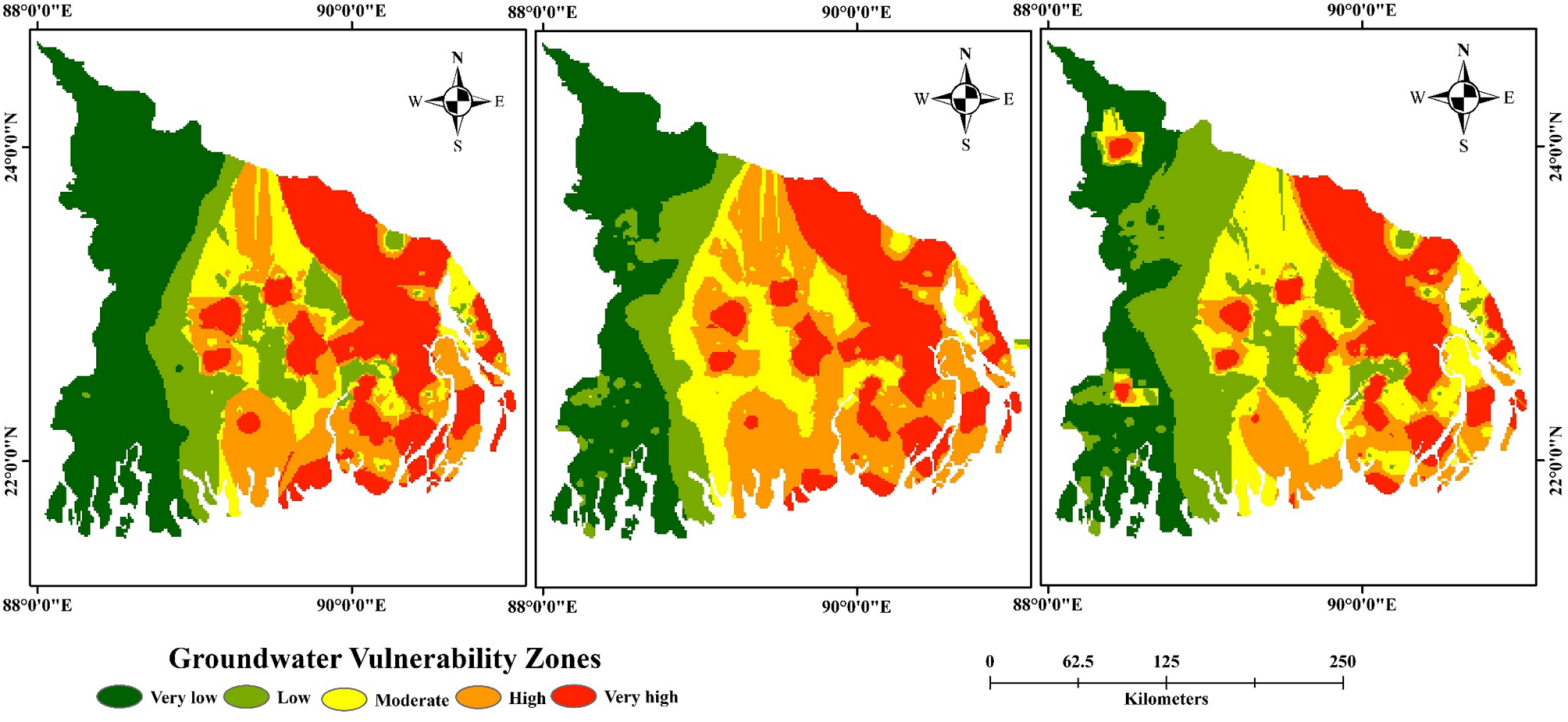
Groundwater vulnerability maps of Ganges delta: (**a**) LR, (**b**) RF and (**c**) ANN.

Groundwater vulnerability in the aquifers of Ganges delta was assessed using LR, RF and ANN models, and the results are presented in Fig. 4. Statistical, ML, and neural network algorithms were used to understand the spatial distribution of groundwater vulnerability in the vulnerable mega-delta region. We used ArcGIS 10.5 software to map the final spatial distribution of vulnerability using the respective modelling outcomes. Each map was classified into five vulnerability zones namely “very low, low, moderate, high and very high” using “Jenk’s natural break method”. The final vulnerability maps show that very high groundwater vulnerability zones are found in the eastern and some isolated south-eastern and central middle portions. Conversely, very low groundwater vulnerability zones are found in the north-western, eastern, and south-western parts. The moderate vulnerability zone is found in the central part and isolated patches of the south-eastern and southern parts of the study area. Due to the high concentration of As and other contaminated factors in the groundwater, the eastern part of the Ganges delta, i.e., the region of Bangladesh, is very vulnerable to groundwater compared to the western part of the delta region, i.e., the state of West Bengal in India. Although two isolated patches are found to be in the very high vulnerable zone in the western region of the Ganges delta i.e., part of India in RF and ANN models (Fig. 4).

### Importance hydrochemical parameters for groundwater vulnerability

It is a fact that all selected hydrochemical parameters have not equal responsibility for groundwater vulnerability assessment in this study. Therefore, it is fundamental to determine the dominant factors in each applied learning model for groundwater vulnerability. The most dominant factors were identified for the three applied models, i.e., LR, RF, and ANN. The results of the dominant factors for groundwater vulnerability are presented in Table 3 for the three applied models. Factors such as F^−^ (0.74), Na^+^ (0.77), As (0.69), Mg^2+^ (0.58) and HCO_3_ (0.54) are more dominant, while SO_4_^2^ (0.2), pH (0.21), EC (0.31) and PO_4_^2^ (0.32) are less dominant in the LR model. The “mean decrease accuracy (MDA) method of RF algorithm” revealed that Na^+^ (0.84), F^−^ (0.77) and As (0.72) are the most influential factors on groundwater resources followed by HCO_3_ (0.55), and Mg^2+^ (0.54). In ANN, the dominant factors are Na^+^ (0.88), F^−^ (0.81), As (0.78) and HCO_3_ (0.67), and the less dominant factors are SO42 (0.19), pH (0.24), salinity (0.31), and EC (0.33).

**Table 3:** Variables importance of selected factors through applied three models.

Factors	LR	RF	ANN
NO_3_	0.44	0.35	0.41
AS	0.69	0.72	0.78
PO_4_^2−^	0.32	0.21	0.38
SO_4_^2−^	0.2	0.22	0.19
Salinity	0.32	0.24	0.31
HCO_3_	0.54	0.55	0.67
CI	0.41	0.38	0.47
K+	0.24	0.25	0.28
Na^+^	0.77	0.84	0.88
Mg^2+^	0.58	0.54	0.51
F^−^	0.74	0.77	0.81
pH	0.21	0.28	0.24
EC	0.31	0.39	0.33
Depth	0.4	0.41	0.41
Ca^2+^	0.33	0.31	0.35

### Evaluation assessment

**Figure 5:**
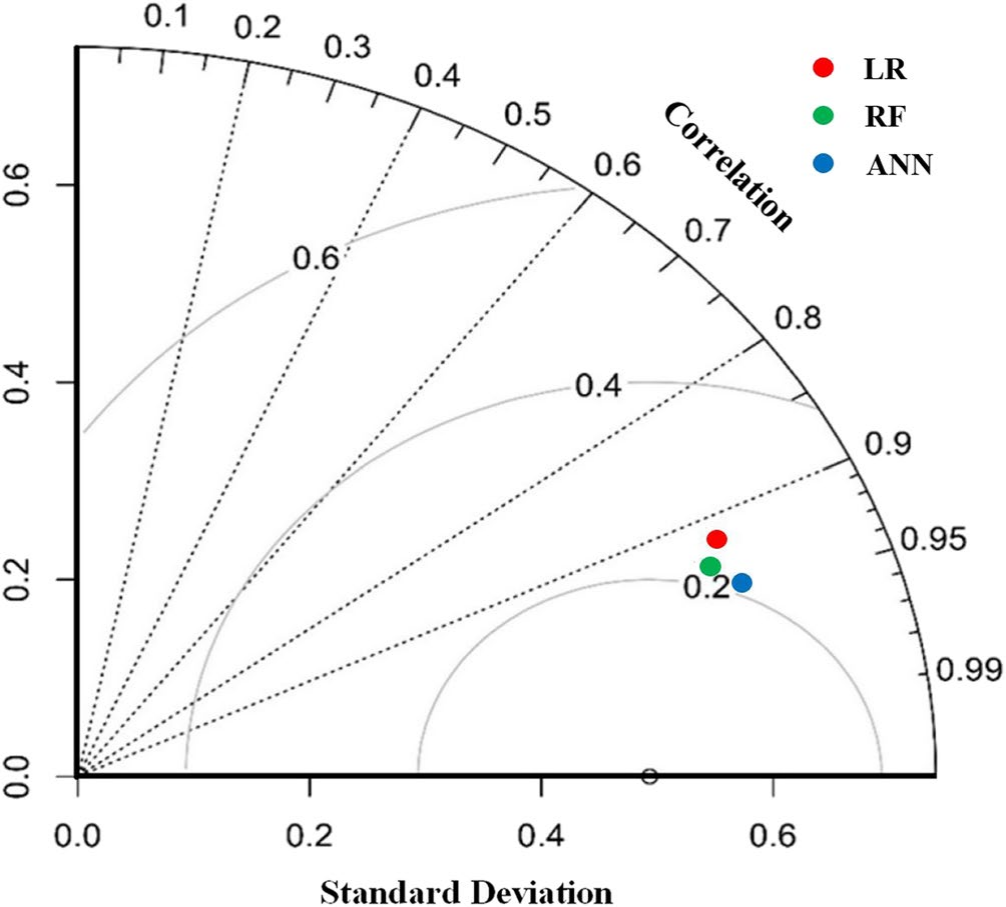
Graphical evaluation measure of applied models using Taylor diagram.

All three models were evaluated using various metrics such as “sensitivity, specificity, NPV, PPV, ROC-AUC, Kappa-coefficient, and F-score”. Among the three models, the ANN model is the most suitable for modelling groundwater vulnerability, with a ROC value of 0.912 and 0.902, for training and validation, respectively. This is followed by the RF model with 0.817 and 0.792 for training and validation, and then the LR model with 0.749 and 0.712 for training and validation. The PPV and NPV are also high in the ANN model, with values of 0.883 and 0.885 in the validation stage. The sensitivity analysis showed that the ANN model had the highest result at 0.889, followed by RF and LR, with 0.782 and 0.721, respectively, in the validation stage. The Kappa and F-score also indicate that the ANN model is the best fit with values of 0.643 and 0.882 in the validation stage, followed by RF and LR (Table 4). The Taylor diagram in Fig. 5 also shows that the ANN is optimal based on standard deviation and correlation.

**Table 4:** Evaluation metrices of applied models.

Models	Stage	Parameters					Kappa	F-Score
		Sensitivity	Specificity	PPV	NPV	AUC		
LR	Train	0.741	0.749	0.752	0.721	0.749	0.532	0.811
	Validation	0.721	0.722	0.711	0.701	0.712	0.513	0.792
RF	Train	0.821	0.812	0.813	0.825	0.817	0.63	0.863
	Validation	0.782	0.751	0.794	0.744	0.792	0.601	0.841
ANN	Train	0.907	0.903	0.901	0.901	0.912	0.651	0.912
	Validation	0.889	0.891	0.883	0.885	0.902	0.643	0.882

### Quality assessment of groundwater

**Figure 6:**
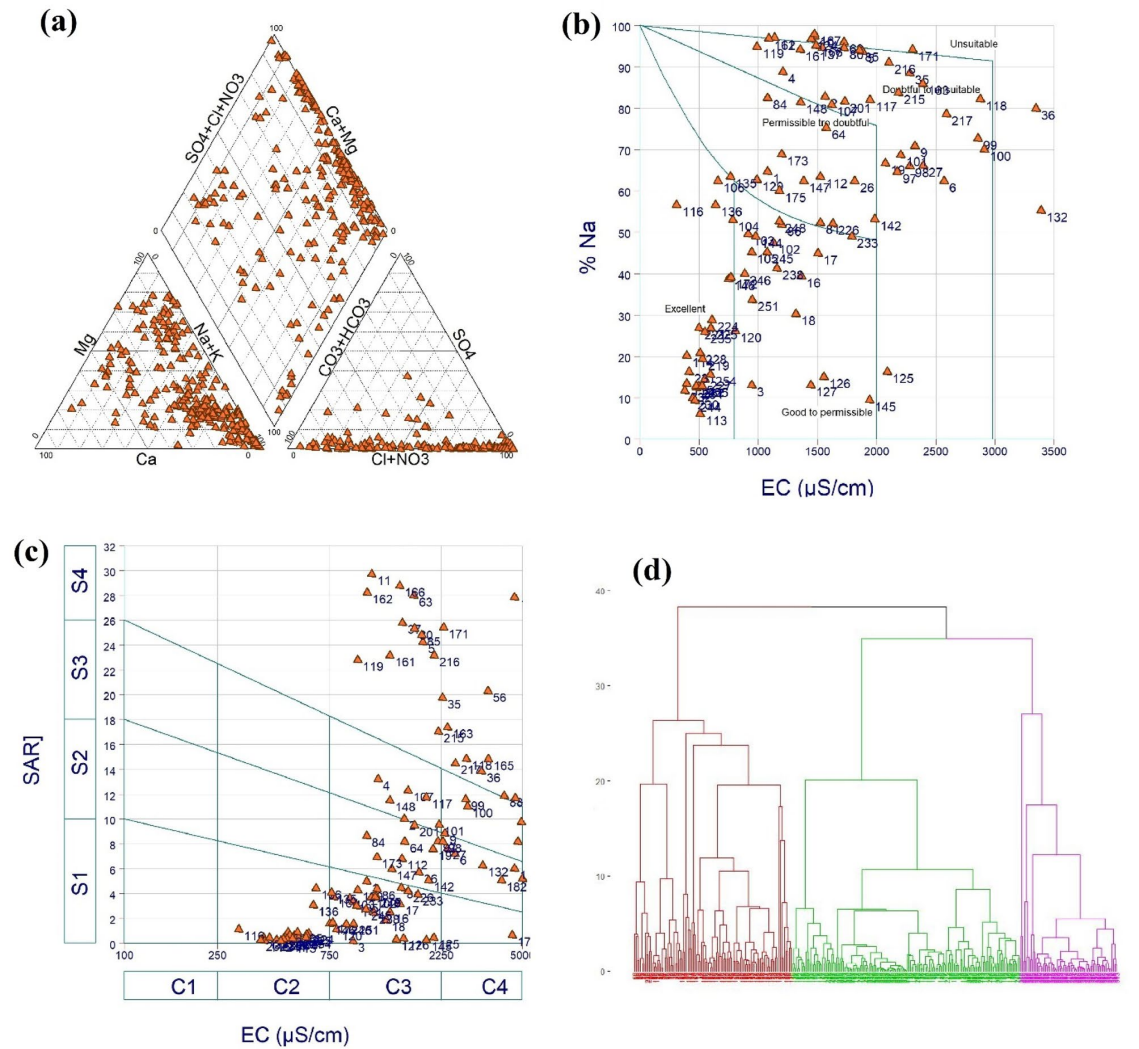
Quality assessment of groundwater: (**a**) Piper diagram, (**b**) Wilcox’s diagram, (**c**) USSL diagram, and (**d**) cluster analysis using dendrogram.

Piper, USSL, and Wilcox diagrams were used to assess the hydrochemical properties and quality of groundwater in the Ganges delta region. The Piper diagram (Fig. 6a) showed that alkaline earth (Ca^2+^+Mg^2+^) dominates over alkalies (Na+K) and that CI and NO_3_ dominate over HCO_3_. The Wilcox diagram (Fig. 6b) showed that two samples were unsuitable, while the others fell into the doubtful to unsuitable, permissible to doubtful, good to permissible and excellent categories. The USSL diagram (Fig. 6c) revealed a high salinity, low sodium and alkali hazard dominance. The collected datasets were grouped and analyzed using a hierarchical clustering method, which showed that the second cluster, and to a lesser extent, the first cluster, significantly influenced the state and the groundwater quality. The dendrogram (Fig. 6d) showed that the first cluster covered approximately 32% of the datasets, the second cluster covered the maximum dataset (47%) and the third cluster covered the lowest dataset (21%).

## Discussion

The fundamental reasons for spatio-temporal fluctuations in the groundwater supply are increasing water demand across all sectors and changing climatic conditions^57^. These factors present a significant challenge to water resource planners. This study has demonstrated that considerable concentrations of elevated arsenic and nitrate in groundwater, as well as salinization, are among the groundwater quality issues in the coastal areas of the multi-aquifers of the Ganges delta. The quality of groundwater along the coast primarily depends on geological conditions, hydrogeological processes, and chemical activities^58^. Therefore, a trustworthy assessment of groundwater vulnerability is a crucial first step in choosing the best design or framework for future water resource development.

It can be challenging to select a preferred model for assessing inherent vulnerability that can effectively match the research topic's features and the study area's geo-environmental characteristics. The literature reveals that many academics have compared two or more vulnerability indices to create a meticulously tailored intrinsic vulnerability model for their research, aiming to achieve optimal output^59,60^. In the current scenario, statistical and machine learning (ML) algorithms are widely employed in groundwater-related studies worldwide. For example, Yu et al. (2022)Vu^61^ applied an integrated Variable Weight Model (VWM) and DRASTIC model to assess groundwater vulnerability in China and found that the VWM-DRASTIC combination provided optimal predictive analysis. Vu et al.^62^ used a numerical model and the index-overlay method in conjunction with climate scenarios (RCPs) to evaluate groundwater vulnerability and associated sustainability in Taiwan, and they recommended optimal predictive analysis. Furthermore, several machine learning models have been utilized in various groundwater-related studies, including groundwater vulnerability^4,14,18,63^, nitrate concentration in groundwater^64–66^, and more. The random forest (RF) model is well-known for its numerous advantages and has been employed in various geoscientific fields, including groundwater vulnerability studies. Lahjouj et al.^67^ utilized the RF algorithm in a survey of groundwater vulnerability to nitrate concentration in Morocco and achieved an accuracy assessment of 0.822 in terms of AUC-ROC. Similarly, Saha et al.^18^ used RF to assess hydrochemical-based groundwater vulnerability in parts of the Ganges delta and achieved optimal accuracy rates of 0.849 and 0.812 in the training and validation data of the ROC. Various statistical techniques are available, ranging from straightforward descriptive statistics of concentrations of specific contaminants to more complex regression analyses that consider the impacts of multiple predictor variables^68^. Binary logistic regression, sometimes known as logistic regression (LR), is a frequently used statistical technique for estimating groundwater vulnerability. LR models relate the potential influencing factors to the likelihood that a pollutant concentration will exceed a threshold value. Mohammaddost et al.^69^ employed DRASTIC, EBF, and LR models in the Kabul basin of Afghanistan to assess groundwater vulnerability, and they found that LR provided 66% accuracy in AUC-ROC prediction analysis. Adiat et al.^70^ applied LR for the same assessment in the Ilesa gold mining area of Nigeria, achieving an 85.7% accuracy in model prediction. Recently, with the significant advantages of neural network algorithms, several neural network models have also been used in groundwater studies. For instance, Elzain et al.^27^ used the DLNN model in aquifer vulnerability studies in South Korea, while Elzain et al.^71^ employed the RBNN model to assess groundwater vulnerability to nitrate contamination in the southern part of Korea.

Based on the discussion above and considering the significant advantages of statistical, machine learning (ML), and neural network algorithms, three popular learning algorithms, namely logistic regression (LR), random forest (RF), and artificial neural network (ANN), were selected for the optimal assessment of groundwater vulnerability in the mega delta of the Ganges delta, taking into account field-based hydrochemical parameters. The findings of this study demonstrate that among the applied models, ANN yields the most optimal results, with AUC-ROC scores of 0.912 and 0.902 in training and validation, respectively, for groundwater vulnerability studies. RF follows with scores of 0.817 and 0.792 in training and validation, and LR with scores of 0.749 and 0.712 in training and validation. The high performance of the ANN model can be attributed to its capacity for parallel processing, enabling it to handle multiple tasks simultaneously. The statistical analysis of all selected hydrochemical parameters reveals that pH and K^+^ (0.952) and EC and Cl^−^ (0.973) are highly correlated, while pH and salinity (0.546), pH and Mg^2+^ (0.506), EC and Na^+^ (0.644), Mg^2+^ and K^+^ (0.593), and Na^+^ and Cl^−^ (0.613) show moderate correlations. It is also found that pH, NO_3_^−^, As, and K^+^ are the most influential factors for groundwater vulnerability in this study region.

Henceforth, studies on groundwater vulnerability serve as crucial measurements for the sustainable management of water resources, environmental preservation, and the guarantee of a secure and uncontaminated drinking water supply for both present and future generations.

Nonetheless, it is a fact that employing combined techniques and methodologies can aid in resolving ambiguities related to GIS-based vulnerability assessment frameworks in geoscientific fields. The approaches presented in this research can be tested in various hydrogeological and geo-environmental contexts to understand the spatial distribution of vulnerability. Evaluating groundwater vulnerability studies requires careful consideration of the data and tools used for validation. Furthermore, the limitations of this study are not considered various important factors, such as the hydrogeological process of groundwater, land use land cover, and aquifer and soil characteristics, as all of these factors affect groundwater quality. In the future, other neural networks and deep learning algorithms can be beneficial for the optimal assessment of groundwater vulnerability in the mega-delta, considering changing climate and land use land cover. Therefore, the results of this study will be valuable to land use planners and provide fundamental information for the optimal assessment and management of groundwater risk zones accordingly.

## Conclusion

Globally, assessing susceptibility to groundwater contamination is crucial for proactive management aimed at safeguarding groundwater resources for various uses. Creating more effective sustainable development policies regarding potential groundwater pollution by utilizing more precise vulnerability maps. In the Ganges deltaic region, the high concentrations of contaminants, such as arsenic (As), are primarily responsible for groundwater vulnerability, and the associated human health hazards are a significant concern for global researchers. In the present research, there is a focus on creating an effective vulnerability map for a mega-delta, specifically the Ganges delta. This involves the application of LR, RF, and ANN models in the modelling and mapping process. Sensitivity analysis indicates that the ANN output is the most optimal, followed by RF and LR. The study reveals that the neural network algorithm is the best suited for assessing groundwater vulnerability related to contamination in the study region, surpassing traditional statistical analysis. Hydrochemical parameters such as pH, NO_3_^−^, As, and K^+^ dominate this deltaic aquifer, contributing to vulnerability. Overall, all vulnerability maps indicate that the study area’s western, central, south, and eastern parts are highly vulnerable. Due to elevated levels of As and various ion contaminations, most groundwater samples from the Ganges delta are unsuitable for drinking and irrigation. Consequently, the improper implementation of government policies, a lack of awareness, and inadequate management are the primary concerns leading to groundwater deterioration in this region. Therefore, immediate action is necessary to sustain and conserve groundwater resources in the world's largest and most densely populated deltaic region. Henceforth, in future application of deep learning and both the dataset i.e., dry and wet season for sampling procedure will be helpful for better understanding of groundwater vulnerability in this vulnerable region.

## Data Availability

The datasets used and/or analyzed during the current study are available from the reasonable request.
